# A Z-Scheme Heterojunction g-C_3_N_4_/WO_3_ for Efficient Photodegradation of Tetracycline Hydrochloride and Rhodamine B

**DOI:** 10.3390/nano15050410

**Published:** 2025-03-06

**Authors:** Yongxin Lu, Shangjie Gao, Teng Ma, Jie Zhang, Haixia Liu, Wei Zhou

**Affiliations:** Shandong Provincial Key Laboratory of Molecular Engineering, School of Chemistry and Chemical Engineering, Qilu University of Technology (Shandong Academy of Sciences), Jinan 250353, China; 10431220336@stu.qlu.edu.cn (Y.L.); 10431220335@stu.qlu.edu.cn (S.G.); 10431231074@stu.qlu.edu.cn (T.M.); zh_jie@qlu.edu.cn (J.Z.)

**Keywords:** g-C_3_N_4_/WO_3_ nanocomposite, photocatalysis, organic pollutants

## Abstract

The construction of heterojunctions can effectively inhibit the rapid recombination of photogenerated electrons and holes in photocatalysts and offers great potential for pollutant degradation. In this study, a Z-scheme heterojunction g-C_3_N_4_/WO_3_ photocatalyst was synthesized using a combination of hydrothermal and calcination methods. The photocatalytic degradation performance was tested under visible light; the degradation efficiency of Rh B reached 97.9% within 15 min and that of TC-HCl reached 93.3% within 180 min. The excellent photocatalytic performance of g-C_3_N_4_/WO_3_ composites can be attributed to the improved absorption of visible light, the increase in surface area, and the effective separation of photogenerated electron–hole pairs. In addition, after four cycles of experiments, the photocatalytic performance of g-C_3_N_4_/WO_3_ did not decrease obviously, remaining at 97.8%, which proved that the g-C_3_N_4_/WO_3_ heterojunction had high stability and reusability. The active radical capture experiment confirmed that h^+^ and ·O_2_^−^ played a leading role in the photocatalytic degradation. The Z-scheme heterojunction g-C_3_N_4_/WO_3_ designed and synthesized in this study is expected to become an efficient photocatalyst suitable for environmental pollution control.

## 1. Introduction

At present, the increasingly serious problems of environmental pollution and energy shortages have become major challenges facing human society. Using solar energy to degrade pollutants saves energy and does not pollute the environment, thereby offering great application prospects [1,2,3,4,5,6,7]. However, traditional photocatalytic materials have some problems, such as low absorption of visible light and rapid recombination of photogenerated electrons and holes, and the preparation of high-performance photocatalysts is still a huge challenge [8,9,10,11]. At present, the commercial photocatalytic crystals on the market are generally wide-band gap crystal material catalysts, such as TiO_2_, which hardly absorb visible light, resulting in low-efficiency solar utilization [12,13]. Therefore, more and more research has been devoted to the study of materials that can effectively photocatalyze the degradation of pollutants under visible light. Tungsten trioxide (WO_3_) is just such a kind of semiconductor photocatalytic material that can absorb visible light energy for catalytic degradation of pollutants [14,15,16].

Tungsten trioxide (WO_3_) shows excellent visible light catalytic performance because of its narrow band gap of about 2.7–2.8 eV, and it also has the advantages of good catalytic stability, excellent photo-corrosion resistance, environmental protection, non-toxicity, and low cost, so its application field is very wide [17,18,19]. However, the photocatalytic performance of unmodified WO_3_ has some limitations due to the low position of the conduction band (CB) of WO_3_ material, which results in low reduction ability of photogenerated electrons and rapid recombination of photogenerated electron-hole pairs [20,21]. It has been pointed out that the Z-scheme system can not only enhance the light-trapping ability of materials and promote the separation of electrons and holes, but it also has strong redox ability [22]. It is helpful to improve the photocatalytic activity of WO_3_ by constructing Z-scheme heterojunction structures, such as ZnFe_2_O_4_/WO_3_ [23], Pt@Cu_2_O/WO_3_ [24], WO_3_/ZnIn_2_S_4_ [25], Ag_3_PO_4_/Ag/WO_3−x_ [26], CdS/WO_3_ [27,28], WO_3_/g-C_3_N_4_/Bi_2_O_3_ [14], Ag/WO_3_/Bi_2_WO_6_ [29], AgI/WO_3_ [30], TiO_2_/RGO/WO_3_ [31], etc.

As a classical semiconductor material, graphite-like carbon nitride (g-C_3_N_4_) has many advantages, such as rich resources, simple synthesis, appropriate band gap width, high physical and chemical stability, and no pollution, and is widely used in the field of photocatalytic degradation [32,33,34,35]. However, due to serious photo-induced electron–hole recombination, a narrow visible light absorption range, and low oxidation potential, the degradation performance of single g-C_3_N_4_ is greatly reduced [36,37,38]. By loading WO_3_ on g-C_3_N_4_ nanosheets to construct a Z-scheme photocatalytic system, the transport of light-induced carriers can be greatly accelerated, and efficient pollutant degradation can be achieved under visible light irradiation.

In this study, g-C_3_N_4_/WO_3_ composite photocatalytic materials with different g-C_3_N_4_ contents were prepared by hydrothermal and calcination methods. The samples were characterized by X-ray diffraction (XRD), transmission electron microscopy (TEM), X-ray photoelectron spectroscopy (XPS), and Fourier transform infrared spectra (FT-IR). The photocatalytic performance was evaluated according to the degradation of Rh B and TC-HCl under visible light irradiation. In addition, the separation mechanism of photogenerated electrons and holes in the g-C_3_N_4_/WO_3_ catalyst was studied via a free radical capture experiment, which reasonably explained the photocatalytic degradation mechanism.

## 2. Experiments

### 2.1. Materials

Sodium chloride (NaCl), sodium tungstate dihydrate (Na_2_WO_4_·2H_2_O), and melamine were purchased from Sinopharm Chemical Reagent Co., Ltd., Shanghai, China. Hydrochloric acid (HCl) was purchased from Yantai Yuandong Fine Chemicals Co., Ltd., Yantai, China. Anhydrous ethanol was purchased from Tianjin Fuyu Fine Chemical Co., Ltd., Tianjin, China. Rhodamine B (Rh B) was purchased from Tianjin Xiensi Biochemical Technology Co., Ltd., Tianjin, China. Tetracycline hydrochloride (TC-HCl) was purchased from Aladdin Chemical Reagent Co., Ltd., Shanghai, China. All reagents were used without further purification.

### 2.2. Preparation of Photocatalyst

#### 2.2.1. Preparation of g-C_3_N_4_

Melamine was added into a 30 mL covered crucible. Then, it was put into a muffle furnace for calcination, the temperature was raised to 550 °C at a heating rate of 3 °C/min, and the material was calcined at 550 °C for 5 h. After cooling to room temperature, the sample was ground into a fine powder and collected for later use.

#### 2.2.2. Preparation of g-C_3_N_4_/WO_3_

**Scheme 1:**
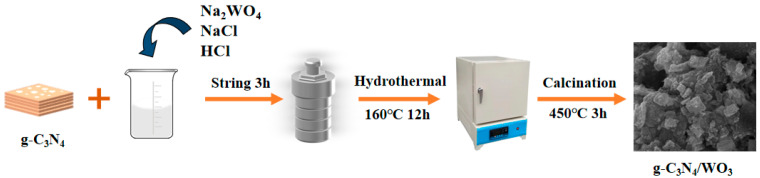
Schematic diagram of the preparation of g-C_3_N_4_/WO_3_.

The g-C_3_N_4_/WO_3_ was synthesized by a facile hydrothermal method and calcination. For this purpose, 4 g of g-C_3_N_4_ was added to 25 mL of deionized water, and ultrasonic treatment was performed for 1 h; then, 0.584 g of NaCl and 1.65 g of Na_2_WO_4_·2H_2_O were added and 5 mL of hydrochloric acid was added to the solution, which was stirred for 3 h until a yellow precipitate appeared. The suspension was transferred to a Teflon-lined autoclave and reacted at 160 °C for 12 h in the oven, cooled to room temperature, filtered, and rinsed to remove impurities, and the obtained sample was dried in an oven at 80 °C for 8 h. Finally, the powder was calcined at 450 °C for 3 h with a heating rate of 5 °C/min to obtain the g-C_3_N_4_/WO_3_. In order to study the influence of g-C_3_N_4_ content on composites, different quantities of g-C_3_N_4_ (0 g, 1.0 g, 2.0 g, 3.0 g, 4.0 g, 5.0 g) were used in the hydrothermal method, and other conditions were kept unchanged. The obtained g-C_3_N_4_/WO_3_ photocatalysts were named WO_3_, g-C_3_N_4_/WO_3_-1, g-C_3_N_4_/WO_3_-2, g-C_3_N_4_/WO_3_-3, g-C_3_N_4_/WO_3_-4, and g-C_3_N_4_/WO_3_-5, respectively. The preparation method is shown in Scheme 1.

### 2.3. Photocatalytic Activities Measurement

First, 0.1 g of the sample was dispersed in 100 mL of Rh B solution (20 mg/L) and sonicated for 5 min to improve the dispersion. The suspension was stirred magnetically in the dark for 30 min to obtain the equilibrium adsorption state before irradiation. Then, 4 mL of the solution was taken out every 5 min under visible light irradiation, and the catalyst was filtered out. The residual concentration of Rh B solution was determined by ultraviolet–visible spectrophotometer (Hitachi UH5300, Tokyo, Japan). In addition, TC-HCl (30 mg/L) was selected as a representative pollutant to study the degradation effect of the catalyst on antibiotics. A 300 W Xe lamp (CEL-HXUV300, Beijing China Education Au-light Co., Ltd., Beijing, China) with a 400 nm filter was used as the light source.

### 2.4. Photoelectrochemical Test

Transient photocurrent response and electrochemical impedance spectroscopy (EIS) were carried out on a CHI 760E electrochemical workstation, and a standard three-electrode system was adopted, including working electrode, counter electrode, and reference electrode. For the measurement, 0.5 M Na_2_SO_4_ was used as the electrolyte solution. First, 0.01 g of catalyst was dissolved in the mixed solution of 0.98 mL absolute ethanol and 0.02 mL Nafion solution (5.0 wt%), and ultrasonic treatment was carried out for more than 1 h to form a uniform slurry. Then, 200 μL of the mixed solution was dripped on the glassy carbon electrode and dried in the air to obtain the working electrode. A 300 W Xe lamp (CEL-HXUV300, Beijing China Education Au-light Co., Ltd., Beijing, China) with a 400 nm filter was used as the light source.

### 2.5. Active Species Capture and Electron Spin Resonance (ESR) Experiment

In the capture experiment of active species, 1 mM ammonium oxalate (AO), 2 mM benzoquinone (BQ), and 10 mM isopropyl alcohol (IPA) were used as scavengers for h^+^, ·O_2_^−^, and ·OH, respectively. In addition, the existence of ·OH and ·O_2_^−^ free radicals was measured by ESR with DMPO as trapping agent. The experimental process was as follows. A 10 mg sample and 40 μL DMPO were dissolved in 0.5 mL deionized water and stirred for 5 min to detect hydroxyl radical (DMPO-·OH). A 10 mg sample and 40 μL DMPO were dissolved in 0.5 mL methanol and stirred for 5 min to detect superoxide radical (DMPO-·O_2_^−^).

### 2.6. Characterization

The powder X-ray diffraction (XRD) patterns of the prepared samples were tested using a Japanese Rigaku smartlab SE X-ray diffractometer (Rigaku Corp, Tokyo, Japan) with a Cu KαX-ray source (λ = 1.5406 Å). The Fourier transform infrared spectra (FT-IR) were collected on a Bruker Tensor FT-IR spectrometer (Billerica, MA, USA). The refined structure and morphology of the samples were recorded via scanning electron microscopy (SEM, TESCAN MIRA LMS instrument operated at 15 kV, Czech Republic, Europe) and transmission electron microscopy (TEM, Talos F200X G2, Waltham, MA, USA, acceleration voltage 200 kV). All the X-ray photoelectron spectroscopy (XPS) measurements of these catalysts were collected with an ESCALABXi + XPS Spectrometer (Waltham, MA, USA). The BET specific surface areas were determined using a Micromeritics ASAP 2460 analyzer (Atlanta, Georgia, USA). The UV-vis diffuse reflectance spectra (DRS) were acquired on a UV2600 spectrophotometer (Shimadzu, Tokyo, Japan). The photoluminescence (PL) spectra were recorded on a Japan Hitachi F4700 fluorescence spectrometer at 350 nm (Tokyo, Japan). The electron spin resonance (ESR) signals were recorded with a Bruker A300 spectrometer (Karlsruhe, Germany).

## 3. Results and Discussion

### 3.1. Photocatalysts Characterization

**Figure 1:**
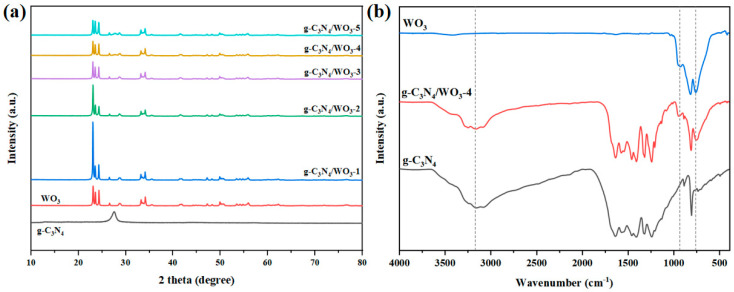
XRD patterns of all prepared samples (**a**), and FTIR spectra of g-C_3_N_4_, WO_3_, and g-C_3_N_4_/WO_3_-4 (**b**).

The crystal phase and crystallinity of g-C_3_N_4_, WO_3_, and g-C_3_N_4_/WO_3_ composites were recorded by XRD. As shown in Figure 1a, the g-C_3_N_4_ sample had two characteristic peaks. The weak peak at 13.2° corresponds to the (100) crystal plane. Due to the interfacial superposition of aromatic systems, the peak at 27.4° corresponds to the (002) crystal plane [39]. Pure WO_3_ exhibits diffraction peaks at 23.1°, 23.6°, 24.4°, 26.6°, 28.6°, 33.3°, 33.6°, and 34.2°, which match well with the (002), (020), (200), (120), (112), (022), (202), and (202) crystal planes of WO_3_ (PDF#83-0951), respectively. All g-C_3_N_4_/WO_3_ composites had XRD patterns similar to pure WO_3_, indicating that WO_3_ was the main component in the g-C_3_N_4_/WO_3_ samples. The (002) diffraction peak position of g-C_3_N_4_ was close to the diffraction peak of the WO_3_ (120) (112) crystal plane. With the increase in g-C_3_N_4_ content, the relative diffraction peak intensity of WO_3_ gradually weakened, and the characteristic peak of g-C_3_N_4_ gradually increased. The characteristic peaks of g-C_3_N_4_ and WO_3_ were detected in the XRD patterns of g-C_3_N_4_/WO_3_, and there were no other miscellaneous peaks. The above results show that g-C_3_N_4_ and WO_3_ existed in the composite material. The chemical states of g-C_3_N_4_, WO_3_ nanosheets, and g-C_3_N_4_/WO_3_-4 composite were studied via FT-IR spectroscopy (Figure 1b). Because the peak shapes of all the composite materials were generally similar, for better and clearer comparison, the figure includes the FT-IR spectrum of only the g-C_3_N_4_/WO_3_-4 composite material. For WO_3_, a broad peak in the range of 500–1000 cm^−1^ is shown, which is attributed to the W-O-W stretching mode. For g-C_3_N_4_ and g-C_3_N_4_/WO_3_-4 samples, there is a typical absorption peak at 809 cm^−1^, corresponding to the functional groups of the triazine structure. Other bands located between 1200 and 1700 cm^−1^ are related to the stretching vibration of C-N heterocycles in the g-C_3_N_4_ material [40]. The g-C_3_N_4_/WO_3_-4 composite material exhibited a spectrum similar to that of pure g-C_3_N_4_. Looking closely at the spectral band of 500–1200 cm^−1^, the broad peaks of WO_3_ can be seen. These results show that the WO_3_ nanosheets were successfully inserted into the g-C_3_N_4_ layers, and there was a strong interaction between them.

**Figure 2:**
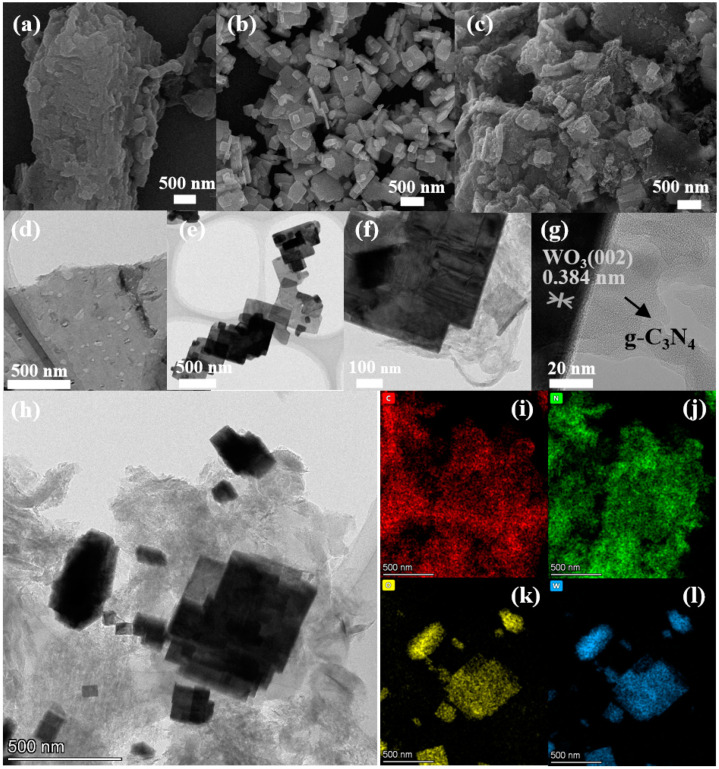
SEM micrographs of (**a**) g-C_3_N_4_, (**b**) WO_3_, (**c**) g-C_3_N_4_/WO_3_-4. TEM, HRTEM results of (**d**) g-C_3_N_4_, (**e**) WO_3_, (**f**,**g**) g-C_3_N_4_/WO_3_-4, (**h**–**l**) EDS images of g-C_3_N_4_/WO_3_-4:C (red), N (green), O (yellow), and W (blue).

The morphology and microstructure of g-C_3_N_4_, WO_3_, and the obtained g-C_3_N_4_/WO_3_-4 composite were analyzed via SEM, TEM, and HRTEM. Because the morphology of all the composite materials was very similar, in order to explain the morphology more clearly, we chose g-C_3_N_4_/WO_3_-4, which had the best catalytic effect, as representative of composite materials. Figure 2a shows that the g-C_3_N_4_ sample presented an aggregated layered stacked structure; flexible nanosheets with large lateral dimensions can be observed, which is a typical structural feature of g-C_3_N_4_ nanosheets. The porous layered structure of g-C_3_N_4_ could be clearly seen via TEM (Figure 2d), and the existence of a porous structure added more active sites to it. In Figure 2b, it can be observed that the prepared WO_3_ was mainly composed of a nanosheet structure with a smooth surface. The HRTEM image of WO_3_ nanosheets (Figure 2e) shows clear lattice fringes with a measured stripe spacing of 0.384 nm, belonging to the (002) crystal plane of monoclinic WO_3_. In the TEM image of the g-C_3_N_4_/WO_3_-4 sample (Figure 2f), the part with higher contrast can be attributed to the WO_3_ nanosheets. On further observation, HRTEM images showed a clear contact interface between WO_3_ and g-C_3_N_4_ (Figure 2g). The clear lattice stripes in the upper part of the image can be attributed to WO_3_. In addition, the composition and structure of the composite were further confirmed by the EDS spectrum and elemental mapping. As shown in Figure 2h–l, C, N, O, and W coexisted in the EDS spectrum of the g-C_3_N_4_/WO_3_-4 composite, and the distribution positions of the elements in the element mapping were the same as those in the TEM image. The interface contact between WO_3_ and g-C_3_N_4_ was very close, benefitting the interface charge transfer between WO_3_ and g-C_3_N_4_ during photocatalysis.

Na_2_WO_4_ + 2HCl → H_2_WO_4_ + 2NaCl (1)

H_2_WO_4_ → WO_3_ + H_2_O (2)

**Scheme 2:**
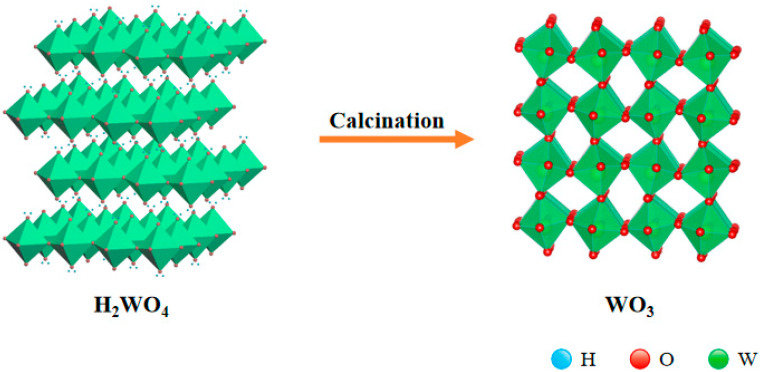
Schematic diagram of crystal structure transformation.

The formation process of the WO_3_ nanosheets was analyzed. After HCl was added dropwise to Na_2_WO_4_ solution, the reaction formed H_2_WO_4_. When the temperature exceeded the decomposition temperature of H_2_WO_4_, WO_3_ started to nucleate and the WO_3_ crystal nucleus was obtained. Transformation from H_2_WO_4_ nanosheets to WO_3_ nanosheets is a topological chemical transformation process (Scheme 2). In its orthogonal crystal form, H_2_WO_4_ is composed of WO_6_ octahedral layers that share four equatorial oxygen atoms to form sheets, and these sheets are linked together by hydrogen bonds between water molecules and oxygen atoms. H_2_WO_4_ easily forms a sheet-like morphology because the layered structure can be separated by water molecules along the (010) plane. When heated to a certain temperature, crystal water is released from H_2_WO_4_, which leads to a change in the crystal structure of H_2_WO_4_. The hydrogen bonds between two adjacent [WO_5_(OH_2_)] octahedral layers are replaced by W-O-W bonds, forming a three-dimensional W-O-W network structure, and the WO_6_ layers are closely combined, thus forming ultra-thin WO_3_ nanosheets by intercalation. After high-temperature calcination, the edges of the nanosheets become smoother, which further improves the photocatalytic activity and efficiency of the WO_3_ nanosheets.

**Figure 3:**
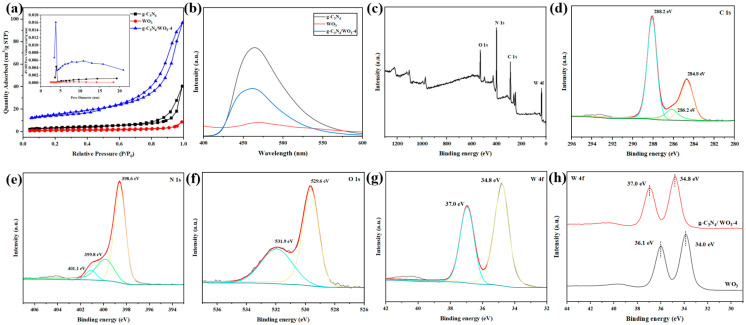
(**a**) N_2_ adsorption and desorption isotherms and pore size distribution curves (inset) of g-C_3_N_4_, WO_3_, and g-C_3_N_4_/WO_3_-4, (**b**) PL spectra of g-C_3_N_4_, WO_3_, and g-C_3_N_4_/WO_3_-4, (**c**) XPS survey spectrum of g-C_3_N_4_/WO_3_-4, high-resolution XPS spectra: (**d**) C 1s, (**e**) N 1s, (**f**) O 1s, (**g**) W 4f, (**h**) XPS of WO_3_ and g-C_3_N_4_/WO_3_-4 in the region of W 4f.

The chemical interaction between g-C_3_N_4_ and WO_3_ was studied via XPS analysis, and the results are shown in Figure 3c–h. The spectrum of g-C_3_N_4_/WO_3_-4 showed that W 4f, O 1s, C 1s, and N 1s coexisted. Next, the peaks of each element were analyzed in detail. The high-resolution C 1s spectrum showed two main peaks at 284.8 eV and 288.2 eV, which could be attributed to the sp^2^ C-C bond and sp^2^ hybrid N-C=N bond, while the weak peak at 286.2 eV may have been caused by incomplete polymerization of the terminal amino groups at the edge of the heptazine unit. The N 1s spectrum showed three peaks located at 398.6 eV, 399.8 eV, and 401.1 eV, respectively, corresponding to C-N=C, N-(C)_3_, and C-N-H. The spectra of W 4f showed two typical peaks, as shown in Figure 3g, which were the W4f_7/2_ and W4f_5/2_ peaks located at 34.8 eV and 37.0 eV, respectively, typical characteristic peaks of W^6+^. The W 4f spectra of WO_3_ and g-C_3_N_4_/WO_3_-4 were compared. It can be seen from Figure 3h that the peak area ratios of W4f_7/2_ and W4f_5/2_ for g-C_3_N_4_/WO_3_-4 and WO_3_ were all 4: 3. The peak of g-C_3_N_4_/WO_3_-4 shifted to a higher binding energy, which may have been caused by the interaction between WO_3_ and g-C_3_N_4_. The increase in binding energy means a decrease in electron density. Therefore, some of the electrons in WO_3_ were transferred to g-C_3_N_4_. The O 1s spectrogram showed two peaks at 529.6 eV and 531.9 eV, corresponding to W-O-W and W-O-H, respectively. 

The specific surface area and pore structure have important effects on the photocatalytic properties of materials. Figure 3a depicts the N_2_ adsorption–desorption curve and pore size distribution of the prepared samples, and calculates the BET surface area and pore volume. It can be seen from the figure that the N_2_ adsorption and desorption curves of all catalysts were type IV isotherms. Pore size analysis revealed that the catalysts were mesoporous. From the Table 1, it can be seen that compared with pure materials, the composite materials exhibited higher specific surface area and pore volume. This may have been due to the formation of WO_3_ nanoplates between the slices of g-C_3_N_4_, causing g-C_3_N_4_ to unfold, thereby increasing its surface area and pore volume. High specific surface area is not only beneficial for improving the adsorption of pollutants on the catalyst, but it also can provide more reactive sites, thus improving the photocatalytic performance of the catalyst [14]. Photoluminescence (PL) testing of the g-C_3_N_4_, WO_3_, and g-C_3_N_4_/WO_3_-4 composites was carried out to study their photoluminescence carrier separation and transfer abilities. Figure 3b shows the photoluminescence spectra of different samples at an excitation wavelength of 350 nm. As shown in the figure, g-C_3_N_4_ showed the highest peak intensity, located at about 470 nm, indicating that g-C_3_N_4_ had the fastest recombination rate of the photogenerated carriers. Compared with g-C_3_N_4_, the peak intensity of g-C_3_N_4_/WO_3_-4 was obviously lower, which indicates that the synthesis of g-C_3_N_4_/WO_3_-4 promotes the efficient separation of electrons and holes in the sample, thus effectively improving the photocatalytic efficiency. Compared with WO_3_, the luminescent intensity of the g-C_3_N_4_/WO_3_-4 composite was higher, which may have been due to the formation of a heterojunction between g-C_3_N_4_ and WO_3_ [41]. When excited by the value of their energy exceeding its band gap, the electrons in the valence bands of g-C_3_N_4_ and WO_3_ all jumped to their conduction bands, and the holes left in the valence band of g-C_3_N_4_ recombined with the electrons in the conduction band of WO_3_, resulting in the peak intensity of the composite being higher than that of WO_3_.

**Figure 4:**
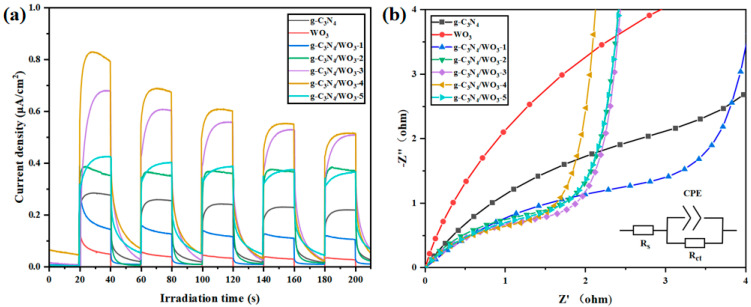
(**a**) Transient photocurrent response tests of all the catalysts and (**b**) EIS plots of all the samples.

In order to study the electron migration rate and separation efficiency of different materials, the transient photocurrent response and electrochemical impedance were tested. Under visible light irradiation, g-C_3_N_4_/WO_3_-4 showed the highest photo current intensity in all samples (Figure 4a). This shows that the g-C_3_N_4_/WO_3_-4 composite had better photogenerated electron–hole separation efficiency than g-C_3_N_4_ and WO_3_. This may have been due to the formation of a heterojunction between g-C_3_N_4_ and WO_3_, which hindered the recombination reaction. Over time, the intensity of the photocurrent decreased slightly, which may have been due to the accumulation of holes. In addition, the migration rate of the samples’ photogenerated charge was also studied via EIS testing (Figure 4b). The radius of the arc is proportional to the charge transfer resistance, and the smaller the radius of the arc, the lower the charge transfer resistance. Equivalent circuits (inset in Figure 4b) were also proposed for further analysis of EIS plots. In the formula, R_s_ is the solution resistance, CPE is the constant phase element, and R_ct_ is the interfacial charge–transfer resistance. As can be seen from Figure 4b, the arc radius of each of the composites was significantly smaller than that of g-C_3_N_4_ or WO_3_. This shows that g-C_3_N_4_/WO_3_-4 exhibited the fastest interfacial charge transfer and the most effective photogenerated charge carrier separation. Therefore, the combination of g-C_3_N_4_ and WO_3_ greatly promoted charge separation, thus improving the photocatalytic efficiency. To investigate the electron transfer rates of different catalysts, we calculated the cyclic voltammetry (CV) curves and the results are shown in Figure S1. The current peak density in the CV curve reflects the electron transfer rate of the measured material; a higher current peak intensity indicates a higher electron transfer rate. From the figure, it can be clearly observed that compared with g-C_3_N_4_ and WO_3_, the CV curve of g-C_3_N_4_/WO_3_-4 contained a larger loop and had a higher photocurrent density. The above results indicate that the recombination of g-C_3_N_4_ and WO_3_ can better inhibit the recombination of photogenerated electrons and holes.

**Figure 5:**
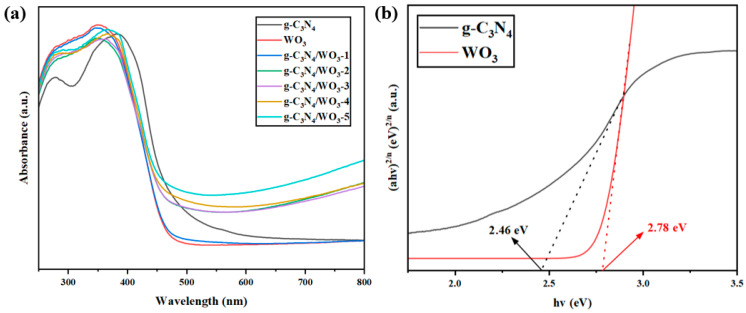
(**a**) UV-vis spectra of all the catalysts and (**b**) the bandgaps of g-C_3_N_4_, WO_3_.

The optical properties of the synthetic catalysts were studied by measuring their UV-vis DRS. As shown in Figure 5a, the absorption edges of g-C_3_N_4_ and WO_3_ appeared at 485 nm and 470 nm, respectively. Compared with g-C_3_N_4_ and WO_3_, the light absorption intensity of the g-C_3_N_4_/WO_3_ composites was obviously enhanced at 500–800 nm, indicating that the combination of g-C_3_N_4_ and WO_3_ was able to utilize more visible light, thus improving the photocatalytic activity.

The band gap width of a photocatalyst can be calculated by the following formula [42]:

αhv = A(hv − Eg)^n/2^ (3)

in which α represents the absorption coefficient, h is Planck’s constant, v is light frequency, and A is a constant. n is related to the type of semiconductor; n is 1 for a direct transition and 4 for an indirect transition. Applying this equation, it can be concluded that the band gaps of g-C_3_N_4_ and WO_3_ are 2.46 eV and 2.78 eV, respectively.

### 3.2. Photocatalytic Degradation Performance

**Figure 6:**
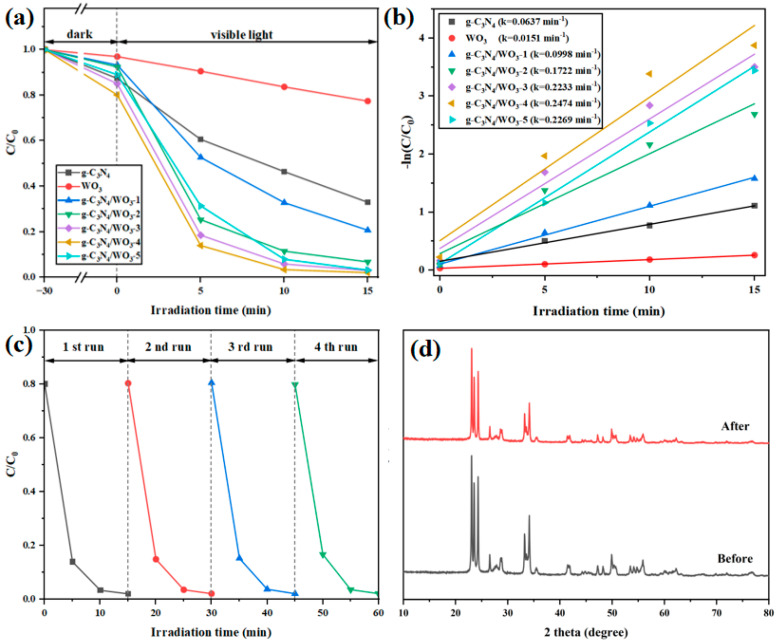
Rh B: [Rh B] = 20.0 mg/L, [catalyst] = 1.0 g/L, pH = 5.7. (**a**) Photocatalytic degradation curves and (**b**) fitted kinetics curves of all the samples, (**c**) cyclic degradation curve of g-C_3_N_4_/WO_3_-4, and (**d**) the XRD pattern of the g-C_3_N_4_/WO_3_-4 sample after 4th run cycle photocatalytic experiments.

The photocatalytic degradation performance of Rh B was tested under visible light, as shown in Figure 6a. After adding the catalyst, the obtained samples were first subjected to dark adsorption for about 30 min, and the 20 mg/L Rh B aqueous solution changed slightly in the dark environment, which was attributed to the self-adsorption of the catalyst. Under visible light irradiation for 15 min, the degradation efficiency of g-C_3_N_4_/WO_3_-4 reached 97.9%. The photocatalytic degradation rate was clearly described using a pseudo-first-order kinetic function. It can be seen from Figure 6b and Table S2 that the g-C_3_N_4_/WO_3_-4 catalyst had the highest photocatalytic degradation rate constant (0.2474 min^−1^). The results show that the content of g-C_3_N_4_ had a great influence on the activity of WO_3_/g-C_3_N_4_ composites, and the optimum content of g-C_3_N_4_ was 4 g. When the amount of added g-C_3_N_4_ was higher than 4 g, the amount of WO_3_ was insufficient to construct an efficient heterojunction between g-C_3_N_4_ and WO_3_ to separate and transfer the photogenerated electron–hole pairs. When the content of g-C_3_N_4_ was too low and the content of WO_3_ was too high, the WO_3_ shell of the composite material became thicker, and the outer layer of WO_3_ was further away from the g-C_3_N_4_. The charge separation became less efficient, and the photogenerated electrons and holes were more likely to recombine during the movement process, reducing the rate of photocatalytic degradation. 

The stability of photocatalysts is crucial in their commercial application. As shown in Figure 6c, the recyclability and stability of g-C_3_N_4_/WO_3_-4 were evaluated. It was observed that the g-C_3_N_4_/WO_3_-4 photocatalyst maintained a degradation efficiency of about 97.8% after four cycles, indicating that g-C_3_N_4_/WO_3_-4 is stable and reusable. XRD testing was conducted on the catalyst after four cycles, and it can be seen from Figure 6d that the XRD patterns before and after degradation were basically consistent, indicating that the crystal structure of g-C_3_N_4_/WO_3_ was not damaged during the catalytic process.

**Figure 7:**
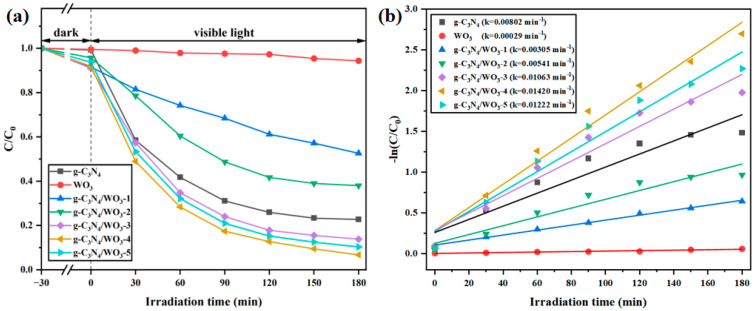
TC-HCl: [TC-HCl] = 30.0 mg/L, [catalyst] = 1.0 g/L, pH = 4.4. (**a**) Photocatalytic degradation curves and (**b**) fitted kinetics curves of all the samples.

In addition to tests with the Rh B dye, degradation experiments were also conducted using the antibiotic tetracycline hydrochloride. As shown in Figure 7 and Table S3, under visible light irradiation, the composite material g-C_3_N_4_/WO_3_-4 exhibited the best photocatalytic degradation performance. At 180 min, the degradation efficiency reached 93.3%. Table S1 summarizes the degradation performance of the g-C_3_N_4_/WO_3_ photocatalyst on organic dyes and antibiotics, indicating that the g-C_3_N_4_/WO_3_ composite exhibits excellent degradation performance compared with other photocatalysts.

**Figure 8:**
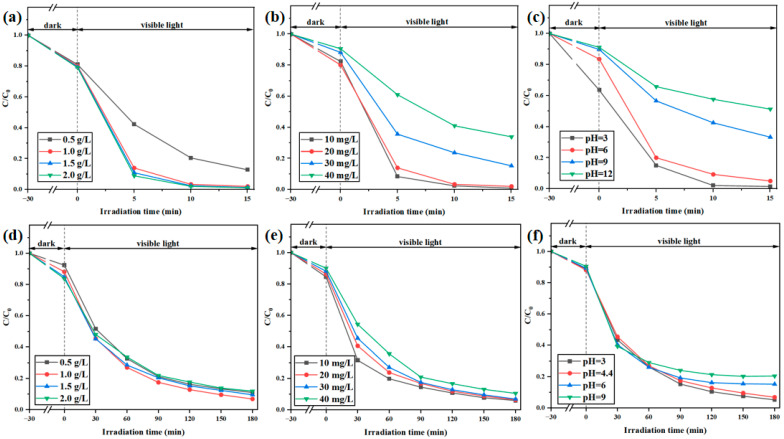
(**a**) Effects of g-C_3_N_4_/WO_3_-4 catalyst dosage on Rh B photodegradation ([Rh B] = 20.0 mg/L, pH = 5.7), (**b**) effects of Rh B initial concentration on the yield of Rh B photodegradation ([catalyst] = 1.0 g/L, pH = 5.7), (**c**) effects of pH on the photocatalytic activity of g-C_3_N_4_/WO_3_-4 in Rh B photodegradation ([Rh B] = 20.0 mg/L, [catalyst] = 1.0 g/L), (**d**) effects of g-C_3_N_4_/WO_3_-4 catalyst dosage on TC-HCl photodegradation ([TC-HCl] = 30.0 mg/L, pH = 4.4), (**e**) effects of TC-HCl initial concentration on the yield of TC-HCl photodegradation ([catalyst] = 1.0 g/L, pH = 4.4), (**f**) effects of pH on the photocatalytic activity of g-C_3_N_4_/WO_3_-4 in TC-HCl photodegradation ([TC-HCl] = 30.0 mg/L, [catalyst] = 1.0 g/L).

In photocatalytic systems, the degradation of pollutants is affected by the surrounding environment. Using Rh B as a pollutant model, the degradation of Rh B was studied under conditions of different pH, catalyst dosage, and pollutant concentration. As shown in Figure 8a, the degradation efficiency increased with the increase in catalyst content, because more catalysts provided more reaction active sites. However, the trend also included weakening due to the excessive addition of catalysts, because when the catalyst content was too high, a suspension was formed, which affected the material’s absorption of light [43]. Figure 8b shows the effects of pollutant concentration on degradation efficiency. As the concentration of pollutants increased, the degradation efficiency gradually decreased. When the catalyst content was constant, the number of active species produced was limited. With the increase in Rh B concentration, dye molecules were adsorbed on the surface of photocatalyst, which reduced the generation of active free radicals [43]. The effects of pH on degradation efficiency are shown in Figure 8c. The isoelectric point of g-C_3_N_4_/WO_3_-4 was determined by the salt addition method [44]. As shown in Figure S3, the pH_iep_ value of the g-C_3_N_4_/WO_3_-4 photocatalyst was found to be 5.29. This indicates that when the pH value was 5.29, the surface charge of the composite was close to zero, and the electrostatic repulsion between particles was at its smallest. In the process of Rh B degradation, the degradation effect was better under acidic conditions, which may have been because when pH < 5.29, the surface of the material was positively charged and the nitrogen-containing groups on the surface of the material were protonated, benefitting to the separation of photogenerated electrons and holes on the surface of the catalyst, thus improving the degradation efficiency of the catalyst [45]. When pH > 5.29 and in an acidic environment, the negatively charged material generated electrostatic attraction with cations in Rh B zwitterions, and the protonation of nitrogen-containing groups remained, so it still had good degradation effect. With the increase in pH, the degradation efficiency decreased gradually, which may have been because at alkaline pH, holes on the surface of g-C_3_N_4_/WO_3_-4 react with OH^−^, resulting in inactivation of photogenerated holes, decreases in active substances, and a decrease in degradation efficiency [46]. Moreover, in alkaline environments, Rh B mainly occurs in basic form and lactone form, which is not conducive to the adsorption of catalysts [47]. Using TC-HCl as a pollutant model, the degradation of TC-HCl was studied under conditions of different pH, catalyst dosage, and pollutant concentration. As shown in Figure 8d, the degradation efficiency first increased and then decreased with the increase in catalyst content, because more catalysts provided more reactive sites. However, when the catalyst content was too high, a suspension was formed, affecting the light absorption of the material. Figure 8e shows the influence of pollutant concentration on degradation efficiency. With the increase in pollutant concentration, the degradation efficiency gradually decreased. When the catalyst content remained constant, the number of active species produced was limited. With the increase in TC-HCl concentration, pollutants were adsorbed on the surface of photocatalyst, which reduced the generation of active free radicals [43]. The effects of pH on degradation efficiency are shown in Figure 8f. From the dark adsorption situation in the first 30 minutes of Figure 8f, it can be seen that the adsorption of TC-HCl by the composite material was almost unaffected by acid-base changes. With the increase in pH, the degradation effect decreased, which may have been due to the better separation of photogenerated electrons and holes under acidic conditions reducing the recombination of photogenerated carriers and producing more active groups [45]. Under alkaline conditions, holes on the surface of g-C_3_N_4_/WO_3_-4 react with OH^−^, resulting in the inactivation of photogenerated holes and a decrease in active substances [46].

### 3.3. Mechanism Considerations

**Figure 9:**
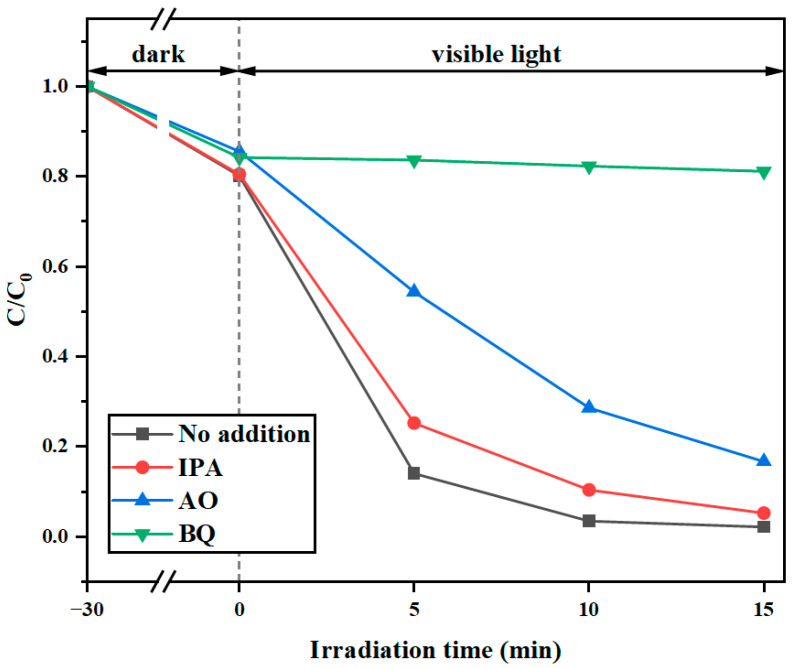
Active species trapping experiments.

In order to study the main active groups in the reaction process, free radical capture experiments were conducted on g-C_3_N_4_/WO_3_-4. The hydroxyl radicals (·OH), holes (h^+^), and superoxide radicals (·O_2_^−^) generated during photocatalytic degradation were quenched using isopropyl alcohol (IPA), ammonium oxalate (AO), and benzoquinone (BQ) radical scavengers. From Figure 9, it can be seen that compared with the situation without inhibitors, the degradation efficiency decreased after adding inhibitors, indicating that ·OH, h^+^ and·O_2_^−^ jointly participated in the photocatalytic degradation of Rh B by g-C_3_N_4_/WO_3_. After adding BQ, the photocatalytic activity of g-C_3_N_4_/WO_3_-4 decreased significantly, and the degradation efficiency was only 22.97%, indicating that ·O_2_^−^ made a significant contribution to the degradation of Rh B in the photocatalytic system. After adding AO, the photocatalytic activity was also inhibited to a certain extent; therefore, h^+^ also played a certain inhibitory role. Compared with the degradation rate of Rh B with the addition of BQ and AO, IPA had the least effect on the degradation rate of Rh B.

**Scheme 3:**
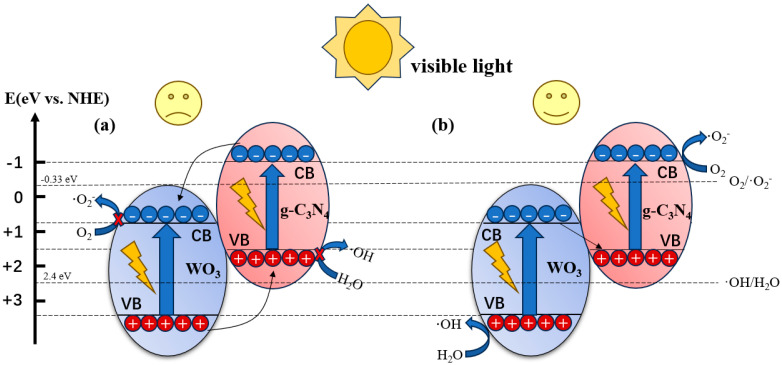
Two possible photocatalytic mechanisms of the g-C_3_N_4_/WO_3_-4 composite, (**a**) Type II mechanism and (**b**) Z-scheme mechanism.

Based on the results of the photocatalytic degradation and active species capture experiments, a possible charge transfer mechanism (Scheme 3) for g-C_3_N_4_/WO_3_ under visible light irradiation was proposed. The valence band (VB) and conduction band (CB) energy positions of a semiconductor can be calculated according to the following formulas:

E(CB) = X-Ee − 0.5Eg(4)

E(VB) = E(CB) + Eg(5)

where X represents the Mulliken’s electronegativity of the semiconductor; the values of X are 6.59 eV and 4.67 eV for WO_3_ and g-C_3_N_4_, respectively. Ee and Eg are the energy of the free electron on the hydrogen scale (about 4.5 eV vs. NHE) and the band gap energy of the semiconductor, respectively. The band gaps of g-C_3_N_4_ and WO_3_ were estimated to be 2.46 eV and 2.78 eV, respectively. According to the calculations, the VB and CB potentials of g-C_3_N_4_ were +1.46 eV and −1.0 eV, respectively, and the VB and CB potentials of WO_3_ were +3.48 eV and 0.7 eV, respectively. The CB and VB positions of g-C_3_N_4_ and WO_3_ were further verified via the Mott–Schottky (MS) method. As shown in Figure S2, the flat-band potentials of g-C_3_N_4_ and WO_3_ were about −1.2 V and +0.5 V (vs. Ag/AgCl), and the CB was 0.1–0.3 eV higher than the flat-band potential. Therefore, the CB potentials of g-C_3_N_4_ and WO_3_ are approximately -1.0 eV and 0.7 eV (vs. NHE). According to the band gap structure of WO_3_ and g-C_3_N_4_, two possible photocatalytic mechanisms of g-C_3_N_4_/WO_3_ were proposed, namely the type II mechanism (Scheme 3a) and the Z-scheme (Scheme 3b). As shown in Scheme 3a, under visible light irradiation, electrons in the CB of g-C_3_N_4_ migrate to the CB of WO_3_, while holes in VB of WO_3_ migrate to the VB of g-C_3_N_4_. Because the calculated CB potential of WO_3_ (0.7 eV vs. NHE) is more positive than the standard redox potential E(O_2_/·O_2_^−^) (−0.33 eV vs. NHE), these electrons accumulated in CB of WO_3_ cannot therefore reduce O_2_ molecules to produce ·O_2_^−^. Because the VB potential of g-C_3_N_4_ (+1.46 eV vs. NHE) is lower than the standard redox potential E(H_2_O/OH^−^) (+2.40 eV vs. NHE), holes in the VB of g-C_3_N_4_ cannot oxidize the H_2_O molecules into ·OH. Therefore, when the charge carrier of the photocatalyst is transferred according to a type II mechanism, it is not conducive to the formation of active species and leads to a decrease in photocatalytic activity within the reaction system. If the charge carriers of the g-C_3_N_4_/WO_3_ photocatalyst are transferred according to the Z-scheme mechanism, as shown in Scheme 3b, the electrons on the CB of WO_3_ are transferred to the VB of g-C_3_N_4_ and finally combined with the photogenerated holes on the VB of g-C_3_N_4_, the remaining holes accumulate in the VB of WO_3_ to generate ·OH, and photogenerated electrons are generated on CB of g-C_3_N_4_ to produce ·O_2_^−^, which is beneficial to the generation of active substances. In addition, the close contact between g-C_3_N_4_ and WO_3_ effectively reduces the migration distance of electrons and holes from the inside to the surface, effectively reduces the migration resistance of the photogenerated carriers, and promotes the rapid migration of those photogenerated carriers. To sum up, Z-scheme mechanism photocatalysts are beneficial to the production of ·O_2_^−^ and ·OH active substances.

In order to further verify the direct Z-scheme mechanism, ESR measurements were used to detect the presence of ·OH and ·O_2_^−^ radicals in the g-C_3_N_4_, WO_3_, and g-C_3_N_4_/WO_3_-4 photocatalytic systems under visible light.

**Figure 10:**
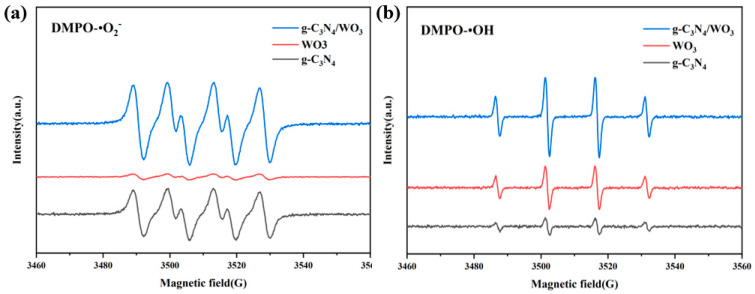
DMPO spin-trapping ESR spectra of g-C_3_N_4_, WO_3_, and g-C_3_N_4_/WO_3_-4 in aqueous dispersion (for DMPO-·OH) (**a**) and in methanol dispersion (for DMPO-·O_2_^−^) (**b**) under visible light irradiation.

As shown in Figure 10a, six characteristic peaks of DMPO-·O_2_^−^ adducts of g-C_3_N_4_ and g-C_3_N_4_/WO_3_-4 were clearly observed, indicating that both samples produced ·O_2_^−^ under illumination. Moreover, the peak strength of g-C_3_N_4_/WO_3_-4 was stronger than that of g-C_3_N_4_, and the g-C_3_N_4_/WO_3_-4 composite produced more ·O_2_^−^. There was almost no peak intensity for WO_3_, consistent with the speculated mechanism. As can be seen from Figure 10b, all three materials had four characteristic peaks of DMPO-·OH adduct with an intensity ratio of 1:2:2:1, which indicates that they all produced ·OH in the photocatalytic reaction. However, analysis of the energy band structure of g-C_3_N_4_ indicates that the valence band of g-C_3_N_4_ cannot directly produce ·OH. Therefore, the ·OH in g-C_3_N_4_ may have been generated by indirect reduction of ·O_2_^−^ [48]. In addition, it can be seen from the figure that the highest ·OH content was produced by g-C_3_N_4_/WO_3_-4, followed by WO_3_. The ESR test results further verify the rationality of the proposed mechanism. The construction of the Z-scheme system retains the outstanding redox ability of g-C_3_N_4_ and WO_3_ and greatly improves the pollutant-degradation abilities of the prepared materials.

## 4. Conclusions

In this study, a Z-scheme g-C_3_N_4_/WO_3_ photocatalyst was successfully prepared by the hydrothermal method with calcination, and its photocatalytic performance was obviously better than that of the pure g-C_3_N_4_ and the WO_3_ materials. After four cycles, the photocatalytic efficiency was almost unchanged, having decreased by only 0.1%, which indicated that the g-C_3_N_4_/WO_3_ photocatalyst had excellent stability. Under visible light irradiation, the degradation rate of Rh B and TC-HCl reached 97.9% and 93.3%, respectively. The constructed Z-scheme system can effectively improve the absorption properties of materials in the visible range, reduce the recombination of photogenerated electron–hole pairs, and retain the outstanding redox abilities of materials. This study demonstrates the rational construction of an efficient Z-scheme composite photocatalyst.

## Figures and Tables

**Table 1:** BET surface areas, pore diameter, and pore volumes of the as-prepared samples.

Photocatalysts	S_BET_ (m^2^ g^−1^)	Pore Diameter (nm)	Pore Volume (cm^3^ g^−1^)
g-C_3_N_4_	11.40	10.81	0.026
WO_3_	4.78	8.66	0.007
g-C_3_N_4_/WO_3_-4	48.72	10.09	0.121

## Data Availability

Data is contained within the article or supplementary material.

## Supplementary Materials

The following supporting information can be downloaded at https://www.mdpi.com/article/10.3390/nano15050410/s1, Table S1: Comparison of photocatalytic degradation of organic dyes and antibiotics by g-C_3_N_4_/WO_3_ catalyst. References [39,49,50,51,52,53] are cited in the Supplementary Materials.
