# Supplementary material for: A Z-Scheme Heterojunction g-C3N4/WO3 for Efficient Photodegradation of Tetracycline Hydrochloride and Rhodamine B
Source: Nanomaterials (Basel). 2025 Mar 6;15(5):410. doi: 10.3390/nano15050410 (PMC11901496; doi:10.3390/nano15050410)
Supplement: Supplementary file 1 [file nanomaterials-15-00410-s001.zip › nanomaterials-3476297-supplementary.pdf]

Supporting Information

A Z-scheme heterojunction g-C_3N_4/WO_3 for efficient photodegradation of tetracycline hydrochloride and rhodamine B

Yongxin Lu,a Jie Zhang,a Shangjie Gao,a Teng Ma,a Haixia Liu,a,* Wei Zhou,a,*

[a] Shandong Provincial Key Laboratory of Molecular Engineering, School of Chemistry and Chemical Engineering, Qilu University of Technology (Shandong Academy of Sciences), Jinan 250353, P.R. China

*Corresponding author. E-mail address: liuhaixia929@163.com; wzhou@qlu.edu.cn

Table. S1 Comparison of photocatalytic degradation of organic dyes and antibiotics by g-C_3N_4/WO_3 catalyst

No.	Catalyst	Dyes	Reaction conditions	Degradation Rate.	Ref.
1	C_3N_4 NP/WO_3 NHMs	TC-HCl	*c*(TC-HCl) = 10 mg/L, *c*(Cat.) = 0.4 g/L, 180 min, Vis light	79.8%	1
2	WO_3/g-C_3N_4 CHMs	TC-HCl	*c*(TC-HCl) = 25 mg/L, *c*(Cat.) = 0.5 g/L, 120 min, Vis light	70%	2
3	RGO/$CdIn_2S_4$/ g-C_3N_4	TC	*c*(TC-HCl) = 10 mg/L, *c*(Cat.) = 1.0 g/L, 180 min, Vis light	74.1%	3
4	g-C_3N_4 nanosheet/WO_3/ graphene oxide	RhB	*c*(RhB) = 10 mg/L, *c*(Cat.) = 0.4 g/L, 60 min, Vis light	97.3%	4
5	Gd/g-C_3N_4/WO_3	RhB	*c*(RhB) = 10 mg/L, *c*(Cat.) = 1.0 g/L, 120 min, Vis light	83.08%	5
6	WO_3/g-C_3N_4	RhB TC-HCl	*c*(RhB) = 10 mg/L, *c*(TC-HCl) = 10 mg/L, *c*(Cat.) = 0.3 g/L, Vis light	100%(RhB, 20 min) 79.1%(TC-HCl, 100 min)	6
7	g-C_3N_4/WO_3	RhB TC-HCl	*c*(RhB) = 20 mg/L, *c*(TC-HCl) = 30 mg/L, *c*(Cat.) = 1.0 g/L, Vis light	97.9%(RhB, 15 min) 93.3%(TC-HCl, 180 min)	Our work

Table. S2 Rate constants, removal rate, and determination coefficients of Rh B degradation by different photocatalysts.

Material	removal rate (%)	K (min^{-1})	determination coefficients (R^2)
$g\text{-}C_3N_4$	67.0	0.0637	0.9967
WO_3	22.6	0.0151	0.9990
$g\text{-}C_3N_4$/ WO_3-1	79.3	0.0998	0.9973
$g\text{-}C_3N_4$/ WO_3-2	93.2	0.1722	0.9610
$g\text{-}C_3N_4$/ WO_3-3	97.0	0.2233	0.9710
$g\text{-}C_3N_4$/ WO_3-4	97.9	0.2474	0.9491
$g\text{-}C_3N_4$/ WO_3-5	96.8	0.2269	0.9943

Table. S3 Rate constants, removal rate, and determination coefficients of TC-HCl degradation by different photocatalysts.

Material	removal rate (%)	K (min^{-1})	determination coefficients (R^2)
$g\text{-}C_3N_4$	77.3	0.0080	0.9087
WO_3	5.6	0.0003	0.9443
$g\text{-}C_3N_4$/ WO_3-1	47.4	0.0031	0.9957
$g\text{-}C_3N_4$/ WO_3-2	62.0	0.0054	0.9360
$g\text{-}C_3N_4$/ WO_3-3	86.2	0.0106	0.9447
$g\text{-}C_3N_4$/ WO_3-4	93.3	0.0142	0.9771
$g\text{-}C_3N_4$/ WO_3-5	89.7	0.0122	0.9598

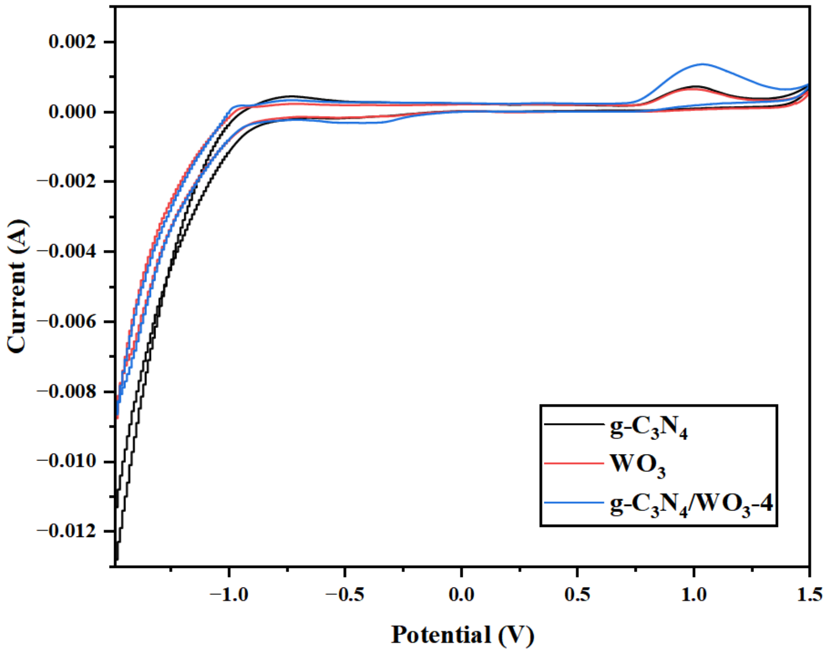

Figure S1. Cyclic voltammetry curve for g-C_3N_4, WO_3, g-C_3N_4/WO_3-4.

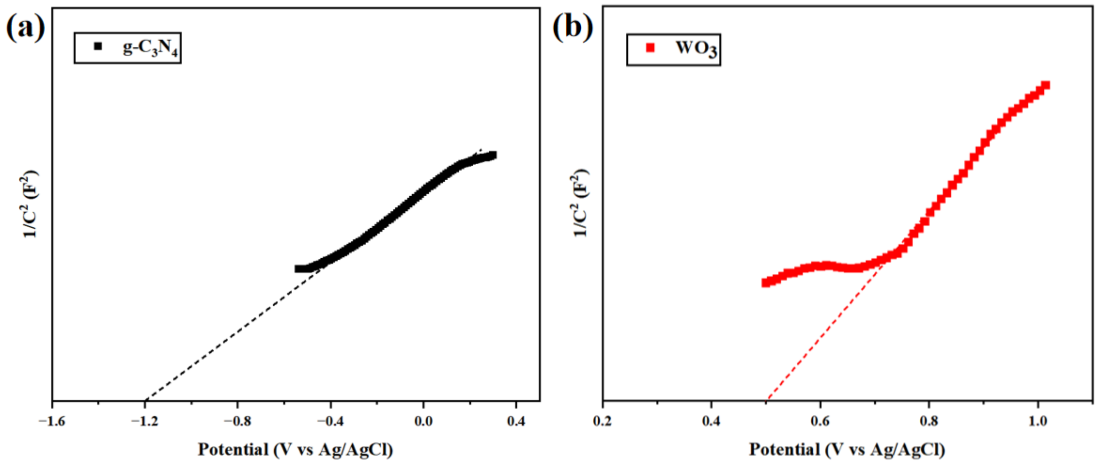

Figure S2. Mott Schottky plot of g-C_3N_4 and WO_3.

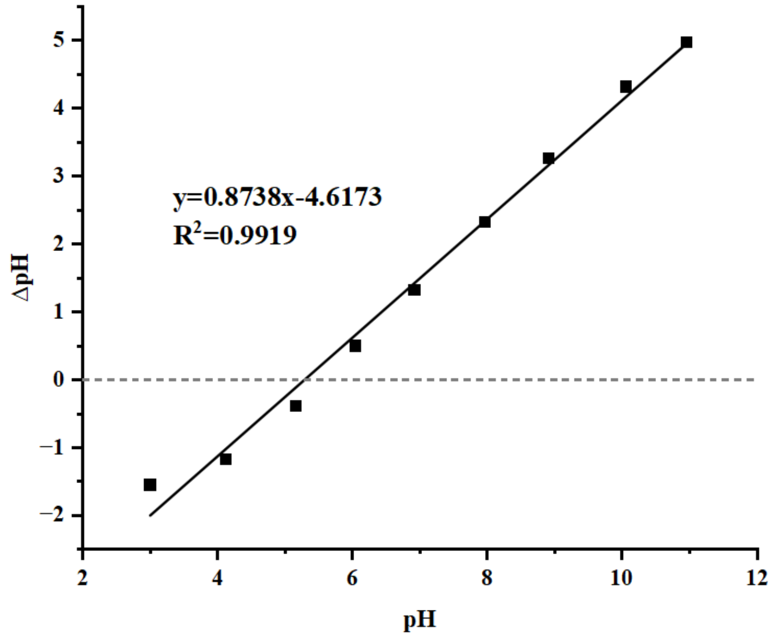

Figure S3. Isoelectric pH determination for g-C_3N_4/WO_3-4 composite.

1. Jing, H.; Ou, R.; Yu, H.; Zhao, Y.; Lu, Y.; Huo, M.; Huo, H.; Wang, X., Engineering of g-C3N4 nanoparticles/WO3 hollow microspheres photocatalyst with Z-scheme heterostructure for boosting tetracycline hydrochloride degradation. *Separation and Purification Technology* **2021,** *255*.

2. Xiao, T.; Tang, Z.; Yang, Y.; Tang, L.; Zhou, Y.; Zou, Z., In situ construction of hierarchical WO3/g-C3N4 composite hollow microspheres as a Z-scheme photocatalyst for the degradation of antibiotics. *Applied Catalysis B: Environmental* **2018,** *220*, 417-428.

3. Xiao, P.; Jiang, D.; Ju, L.; Jing, J.; Chen, M., Construction of RGO/CdIn 2 S 4 /g-C 3 N 4 ternary hybrid with enhanced photocatalytic activity for the degradation of tetracycline hydrochloride. *Applied Surface Science* **2018,** *433*, 388-397.

4. Du, Y.; Zhao, Q.; Liu, R.; Jiang, T., Preparation of g-C3N4 Nanosheet/WO3/Graphene Oxide Ternary Nanocomposite Z-scheme Photocatalyst with Enhanced Visible Light Photocatalytic Activity. *Journal of Cluster Science* **2022,** *34* (1), 273-283.

5. Kalidasan, K.; Mallapur, S.; Kulkarni, B. B.; Maradur, S. P.; Kumar, D.; Deeksha, R.; Kandaiah, S.; Vishwa, P.; Kumar, S. G., Gadolinium modified g-C3N4 for S-Scheme heterojunction with monoclinic-WO3: Insights from DFT studies and related charge carrier dynamics. *Journal of Materials Science & Technology* **2025,** *204*, 166-176.

6. Li, Y.; Wang, J., 2D/2D Z-scheme WO3/g-C3N4 heterojunctions for photocatalytic organic pollutant degradation and nitrogen fixation. *Materials Advances* **2024,** *5* (2), 749-761.
